# Obesity is associated with increased severity of disease in COVID-19 pneumonia: a systematic review and meta-analysis

**DOI:** 10.1186/s40001-020-00464-9

**Published:** 2020-12-02

**Authors:** Yanan Chu, Jinxiu Yang, Jiaran Shi, Pingping Zhang, Xingxiang Wang

**Affiliations:** 1 Department of Cardiology, The First Affiliated Hospital, School of Medicine, Zhejiang University, No. 79 Qingchun Road, Hangzhou, 310006 Zhejiang China; 2 Department of Cardiology, Jinyun People’s Hospital, No. 299 North Ziwei Road, Jinyun, 321400 Zhejiang China

**Keywords:** COVID-19, Obesity, Poor outcomes, Meta-analysis

## Abstract

**Background:**

Obesity has been widely reported to be associated with the disease progression of coronavirus disease 2019 (COVID-19); however, some studies have reported different findings. We conducted a systematic review and meta-analysis to investigate the association between obesity and poor outcomes in patients with COVID-19 pneumonia.

**Methods:**

A systematic review and meta-analysis of studies from the PubMed, Embase, and Web of Science databases from 1 November 2019 to 24 May 2020 was performed. Study quality was assessed, and data extraction was conducted. The meta-analysis was carried out using fixed-effects and random-effects models to calculate odds ratios (ORs) of several poor outcomes in obese and non-obese COVID-19 patients.

**Results:**

Twenty-two studies (*n* = 12,591 patients) were included. Pooled analysis demonstrated that body mass index (BMI) was higher in severe/critical COVID-19 patients than in mild COVID-19 patients (MD 2.48 kg/m^2^, 95% CI [2.00 to 2.96 kg/m^2^]). Additionally, obesity in COVID-19 patients was associated with poor outcomes (OR = 1.683, 95% CI [1.408–2.011]), which comprised severe COVID-19, ICU care, invasive mechanical ventilation use, and disease progression (OR = 4.17, 95% CI [2.32–7.48]; OR = 1.57, 95% CI [1.18–2.09]; OR = 2.13, 95% CI [1.10–4.14]; OR = 1.41, 95% CI [1.26–1.58], respectively). Obesity as a risk factor was greater in younger patients (OR 3.30 vs. 1.72). However, obesity did not increase the risk of hospital mortality (OR = 0.89, 95% CI [0.32–2.51]).

**Conclusions:**

As a result of a potentially critical role of obesity in determining the severity of COVID-19, it is important to collect anthropometric information for COVID-19 patients, especially the younger group. However, obesity may not be associated with hospital mortality, and efforts to understand the impact of obesity on the mortality of COVID-19 patients should be a research priority in the future.

## Introduction

In early December 2019, the rapid propagation of a novel coronavirus broke out in Wuhan, Hubei, China, and caused a highly infectious serious acute respiratory syndrome named coronavirus disease 2019 (COVID-19) [1]. COVID-19 causes high morbidity and mortality worldwide, and the World Health Organization (WHO) officially declared it a pandemic [2]. By 25 May 2020, COVID-19 had caused 5,941,223 confirmed cases and 366,601 related fatalities worldwide [3]. Severe acute respiratory syndrome coronavirus 2 (SARS-CoV-2), which has been identified as the pathogen of COVID-19, is a novel enveloped RNA beta-coronavirus that shares similar genetic identity with two bat-derived coronavirus strains, bat-SL-CoVZC45 and bat-SL-CoVZXC21 [4]. In addition, molecular modelling showed structural similarity between the receptor-binding domains of SARS-CoV and SARS-CoV-2; therefore, SARS-CoV-2 might use angiotensin-converting enzyme 2 (ACE2) as a cell receptor [4]. The affinity of SARS-CoV-2 for ACE2 is approximately 10–20-fold higher than that of SARS-CoV. In addition, ACE2 expression is not limited to the lung; ACE2 is also found in many extrapulmonary organs, such as the oral epithelium, adipose tissue, and heart, which could explain the higher infectivity and multiple organ dysfunction of SARS-CoV-2 infection [5, 6]. Although the majority of COVID-19 patients are asymptomatic or present with mild symptoms such as fatigue and cough, several patients will develop severe or critical pneumonia, characterized as acute respiratory distress syndrome (ARDS), multiorgan failure, and even death [7]. Therefore, more efforts should be directed towards identifying populations at high risk for developing severe or critical COVID-19 [8].

Although several factors have been clearly identified that contribute to the development of severe COVID-19, such as increasing age, male sex, geographic region, and multiple chronic comorbidities, obesity is emerging as an important risk factor, especially in industrialized countries [9]. During the 2009 influenza A virus (IAV) H1N1 pandemic, obesity was linked to an increased risk of severe disease and was a significant risk factor for hospitalization and death [10]. Louie et al*.* [11] reported that over half of hospitalized patients infected with H1N1 were obese, and most deaths occurred in patients who were morbidly obese. A meta-analysis identified 22 articles and indicated that obesity significantly increased the risk of death and critical complications of H1N1 infection [12]. Apart from the evidence from the H1N1 influenza experience, obese subjects with influenza shed the virus for a longer period of time than lean subjects, which increased the transmitting ability of the virus [13]. In addition, the reduced and delayed capacity of producing interferons allows more viral RNA replication, increasing the possibility of novel viral strains, and the unfavourable hormone milieu of obese patients also leads to defects in innate immunity and B and T cell responses [14]. Consequently, emerging evidence indicates an association between obesity and the severity of respiratory infectious diseases. Notably, one French study proposed a higher frequency of obesity among intensive care unit (ICU) patients with SARS-CoV-2-related pneumonia [15]. Similarly, a retrospective case–control study of young Chinese patients with COVID-19 indicated that obesity was the most important critical factor contributing to their death [16]. Peng et al. [17] showed that, in comparison with survivors, non-survivors of COVID-19 patients had a higher body mass index (BMI). However, research regarding fatalities in Italy associated with the COVID-19 pandemic failed to mention obesity as one of the pre-existing diseases associated with death [18]. Previous studies have demonstrated an “obesity paradox”, or an inverse relationship between obesity and mortality among critically ill patients, including those with ARDS [19, 20]. The effects of COVID-19 on patients with obesity have not yet been well described. The latest systemic review emphasized that obesity was a risk factor for the prognosis of COVID-19 [21]. However, the review merely summarized three studies, and more data are needed to support the conclusions. Recently, updated studies assessing obesity and severe COVID-19 have become available, which have amplified the number of obese COVID-19 patients for whom data are available to a large extent. Due to the conflicting evidence, limitations of past reviews, and availability of new data, this study aimed to investigate the association between obesity and poor outcomes of COVID-19 patients by performing a systematic review and meta-analysis.

## Methods and materials

### Search strategy and selection criteria

The study was carried out according to the Meta-analysis of Observational Studies in Epidemiology (MOOSE) guidelines [22] (Additional file 1: Table S1). We carried out a comprehensive electronic search in the PubMed, Embase, and Web of Science databases (Additional file 1: Table S2). Moreover, due to the rapid evolution of information during the current pandemic, preprint articles published on medRxiv (https://www.medrxiv.org) and SSRN (https://www.ssrn.com) were also included, and the reference lists of all potential studies were retrieved to identify additional suitable articles. No language restrictions were applied. Articles published between November 1, 2019, and May 24, 2020, were eligible for inclusion. The following search terms were used: (a) (“2019 novel coronavirus disease” OR “COVID19” OR “COVID-19 pandemic” OR “SARS-CoV-2 infection” OR “COVID-19 virus disease” OR “2019 novel coronavirus infection” OR “2019-nCoV infection” OR “coronavirus disease 2019” OR “coronavirus disease-19” OR “2019-nCoV disease” OR “COVID-19 virus infection” OR “COVID-19”) AND (“Obesity” [MeSH]); and (b) (“2019 novel coronavirus disease” OR “COVID19” OR “COVID-19 pandemic” OR “SARS-CoV-2 infection” OR “COVID-19 virus disease” OR “2019 novel coronavirus infection” OR “2019-nCoV infection” OR “coronavirus disease 2019” OR “coronavirus disease-19” OR “2019-nCoV disease” OR “COVID-19 virus infection” OR “COVID-19”) AND “Characteristics”.

### Inclusion and exclusion criteria

We included all studies that reported (1) information on BMI or the presence of obesity (the WHO defines obesity as BMI ≥ 30 kg/m^2^, while in Asia, obesity is defined as BMI ≥ 28 kg/m^2^) [23, 24] and (2) the specific outcomes of COVID-19 patients, such as mortality, severe COVID-19, ICU care, the usage of invasive mechanical ventilation (IMV), and disease progression of COVID-19. If there were two or more studies from the same authors, only the study with the largest sample size was chosen. Studies with insufficient data to be analyzed, such as review articles, non-research letters, animal experiments, commentaries, meta-analyses, and case series, were excluded.

### Study selection and data extraction

Duplicates were removed. The title and abstract were screened by two independent investigators. The full text of the remaining studies was reviewed according to the inclusion and exclusion criteria. For each study, the following relevant information was extracted: first author, publication time, study design, country, the number of COVID-19 patients, male percentage, the ratio of severe cases to total cases, the prevalence of obesity, BMI, mean or median age of patients, and outcomes. The poor composite outcomes included severe COVID-19, need for ICU care, IMV, mortality, and disease progression. Disease progression was defined when the patient’s condition worsened in terms of at least two poor outcomes mentioned in the included studies. The diagnosis of COVID-19 complied with the WHO interim guidance [25] and the guidelines of the COVID-19 Diagnosis and Treatment Protocol (5th edition) by the National Health Commission of the People’s Republic of China [26]. According to the COVID-19 Diagnosis and Treatment Protocol [26], mild cases were defined as moderate clinical symptoms absent of typical pneumonia changes on CT scan. Severe cases were diagnosed as follows: (a) respiratory distress with a respiratory frequency ≥ 30 breaths/min, (b) a pulse oximeter oxygen saturation ≤ 93% at rest, and (c) an oxygenation index (arterial partial pressure of oxygen/inspired oxygen fraction, PaO_2_/FiO_2_)  ≤ 300 mmHg. Critical cases were defined as (a) respiratory failure requiring mechanical ventilation, (b) the occurrence of shock, and (c) functional failure of other organs and the need for ICU care.

### Assessment of methodological quality and risk of bias

The Newcastle–Ottawa Scale (NOS) was used to assess the quality of the included articles [27] (Additional file 1: Table S3). The NOS is a validated scale for non-randomized studies in meta-analyses. The quality of studies was assessed in three domains: selection, comparability, and outcome/exposure. A study was considered of good quality if there were 3 or 4 stars in the selection domain, 1 or 2 stars in the comparability domain, and 2 or 3 stars in the exposure/outcome domain. If a meta-analysis included more than three studies, the possible publication bias was assessed by a funnel plot, Egger’s test [28], and the trim-and-fill method [29]. Egger’s test *P* values greater than 0.05 indicated no obvious publication bias in this meta-analysis.

### Statistical analysis

A meta-analysis was performed, with calculations of the mean difference (MD) and 95% confidence interval (95% CI) of the BMI of COVID-19 patients. Summary odds ratios (ORs) with 95% CIs were estimated for the association between obesity and poor composite outcomes of COVID-19. Data were assembled and analyzed by Review Manager (RevMan) Version 5.3 (The Nordic Cochrane Center, Copenhagen, Denmark) and Stata 15.0 software. The results were performed with a fixed-effects model (Mantel–Haenszel method) or random-effects model in cases of significant heterogeneity among studies. Heterogeneity among studies was evaluated with the *I*^2^ statistic: 25%, 50%, and 75% represented low, moderate, and high degrees of heterogeneity, respectively. The choice of a proper effect model was based on the analysis results: if significant heterogeneity was not present (*I*^2^ ≤ 50%), we used a fixed-effects model to pool outcomes; a random-effects model was utilized if *I*^2^ > 50%. If there was statistical heterogeneity, further sensitivity analyses were performed by excluding studies one by one. After the studies with significant clinical heterogeneity were excluded, the fixed-effects model was used for meta-analysis. If only the median and interquartile range (Q25, Q75) were reported, we used the method of Wan et al. [30] and McGrath et al. [31] to estimate the mean and standard deviation of the included studies. In the meta-analysis, we compared the risk of poor composite outcomes of COVID-19 patients with and without obesity [including normal-weight and overweight individuals (WHO defines overweight as BMI from 25 to 30 kg/m^2^, while in Asia, overweight is defined as BMI from 24.0 to 27.9 kg/m^2^)]. Random-effects meta-regression was performed using restricted-maximum likelihood for prespecified variables, including age, hypertension, diabetes, cardiovascular disease, and chronic obstructive pulmonary disease (COPD). Then, subgroup analyses were conducted based on poor composite outcomes and mean age (≥ 60 and < 60 years). *P* < 0.05 was considered statistically significant.

## Results

### Study selection

**Fig. 1:**
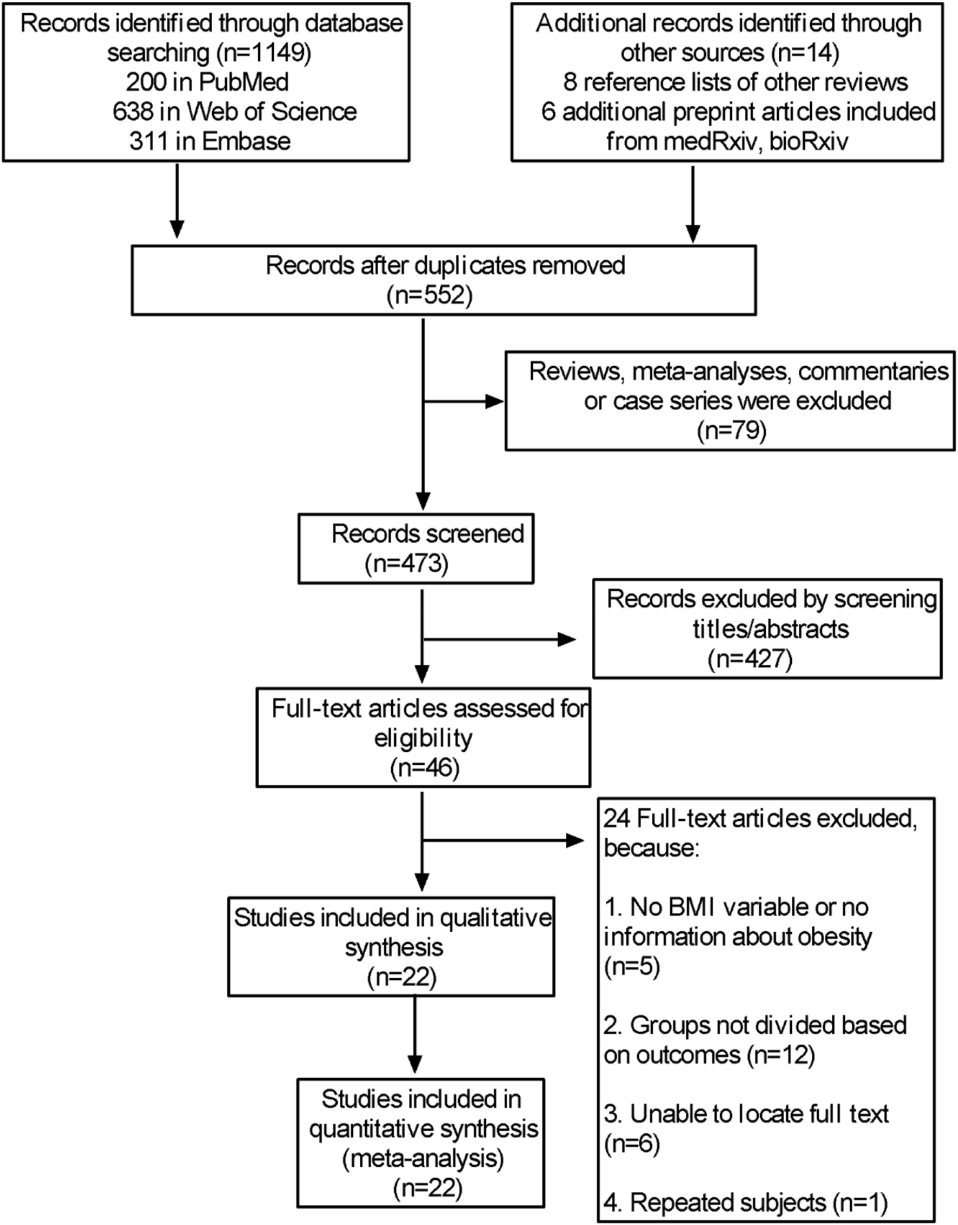
Flow diagram of study selection process. COVID-19: coronavirus disease 2019

In the initial search, 1163 studies were found in different databases; of these, 611 were unique records, and 427 records were excluded after screening their titles and abstracts. We assessed the eligibility of 46 full-text papers, 5 of which did not report BMI or information about obesity, 12 did not divide groups by outcomes, 6 did not have the full text available, and 1 had the same subjects as another article. Figure 1 shows the search details. After excluding these articles, 22 records with 12,591 patients met our prespecified inclusion criteria and were included in the final analysis.

### Description of studies

A summary of the characteristics of each study, including the number of participants per study, country, male percentage, ratio of severe cases to total cases, prevalence of obesity, BMI, and poor composite outcomes, is provided in Table 1. Of the 22 studies that were published between March 2, 2020, and May 23, 2020, over half were from China (12 studies) (Table 1), including a total of 12,591 patients, with sample sizes ranging from 16 [32] to 5279 [33]. The mean age of the patients varied from 24.38 to 68.02 years (median 60.53 years; 22 studies). The proportion of male patients ranged from 33.33 to 75.00% (median 57.43%; 22 studies). The proportion of patients diagnosed with severe/critical COVID-19 varied from 11.39 to 68.55% (median 28.35%; 22 studies). The proportion of patients with obesity ranged from 4.02 to 68.18% (median 35.74%; 16 studies). Two publications were letters [34, 35], and the remainder were journal articles. Nine studies used the WHO criteria to define obesity (Table 1) [15, 32, 33, 35–40].

**Table 1:** Summary of characteristics of the included studies

Study	Country	Publication date	Enrolment duration	Study design (RCS/PS)	Sample size	Male patients (%)	Age, years	BMI cut off (kg/m^2^)	Severe cases/total cases (%)	Severe cases among obese patients/total cases among obese patients (%)	BMI (kg/m^2^) all	BMI (kg/m^2^) severe	BMI (kg/m^2^) non-severe	Primary outcomes
Aggarwal et al. [32]	USA	Apr-29	NA	RCS	16	12 (75.00)	Median 67 (IQR 38–95)	30	8/16 (50.00)	5/8 (62.50)	NA	NA	NA	Disease progression
Argenziano et al. [37]	USA	May-7	Mar 1–Apr 5	RCS	850	511 (60.12)	63.44	30	236/850 (27.76)	107/323 (33.13)	NA	29.4 (25.7–34.2)	28.3 (25.0–32.7)	ICU care
Auld et al. [41]	USA	Apr-26	Mar 6–Apr 17	RCS	217	119 (54.84)	Median 64 (IQR 54–73)	30	62/217 (28.57)	1/21 (4.76)	30 (26–35)	29 (26–32)	31 (27–36)	Death
Buckner et al. [40]	USA	May-22	Mar 2–Mar 26	RCS	105	53 (50.48)	Median 69 (range 23–97)	30	51/105 (48.57)	23/44 (52.27)	NA	NA	NA	Disease progression
Cao et al. [42]	China	Mar-13	Jan 3–Feb 1	PS	102	53 (52.00)	Median 54 (IQR 37–67)	NA	17/102 (16.67)	NA	24.4 (21.8–26.0)	26.0 (23.4–28.7)	24.3 (21.8–25.7)	Death
Cai et al. [49]	China	May-14	Jan 11–Feb 21	PS	383	183 (47.78)	48.73	28	91/383 (23.76)	16/41 (39.02)	NA	NA	NA	Disease progression
Gao et al. [47]	China	Mar-17	Jan 23–Feb 2	RCS	43	26 (60.47)	Mean 43.74 (SD 12.12)	NA	15/43 (34.88)	3/4 (75.00)	NA	NA	NA	Severe COVID-19
Gao et al. [48]	China	May-14	Jan 17–Feb 11	PS	150	94 (62.7)	Mean 48	28	36/150 (24)	25/75 (33.33)	NA	NA	NA	Severe COVID-19
Giacomelli et al. [38]	Italy	May-6	Feb 21–Mar 19	PS	233	161 (69.10)	Median 61 (IQR 50–72)	30	48/233 (20.60)	13/38 (34.21)	NA	NA	NA	Death
Goyal et al. [35]	USA	Apr-17	Mar 3–Mar-27	RCS	393	238 (60.56)	Median 62.2 (IQR 48.6–73.7)	30	130/393 (33.08)	56/136 (41.18)	NA	NA	NA	IMV
Hu et al. [50]	China	May-3	Jan 8–Feb 20	PS	323	166 (51.39)	Median 61 (range 23–91)	30	172/323 (53.25)	8/13 (61.54)	NA	NA	NA	Disease progression
Huang et al. [43]	China	May-8	Jan 22–Feb 10	RCS	202	116 (57.43)	Median 44 (IQR 33–54)	28	23/202 (11.39)	8/24 (33.33)	24.4 (22.3–26.4)	26.4 (23.7–29.5)	24.2 (22.1–26.1)	Severe COVID-19
Klang et al. [36]	USA	May-23	Mar 1–May 17	RCS	3406	1961 (57.57)	66.00	30	1136/3406 (33.35)	384/1231 (31.19)	NA	NA	NA	Death
Kalligeros et al. [39]	USA	Apr-30	Feb 17–Apr 5	RCS	103	63 (61.17)	Median 60 (IQR 50–72)	30	44/103 (42.72)	25/49 (51.02)	NA	NA	NA	ICU care
Peng et al. [17]	China	Mar-2	Jan 20–Feb 15	RCS	112	53 (47.32)	Median 62 (IQR 55–67)	NA	16/112 (14.29)	NA	22.0 (20.0–25.0)	25.5 (23.0–27.5)	22.0 (20.0–24.0)	Critical COVID-19
Liu et al. [44]	China	Mar-12	Feb 10–Feb 31	RCS	30	10 (33.33)	Mean 35 (SD 8)	NA	4/30 (15.38)	NA	NA	27.0 ± 2.5	22.0 ± 1.3	Severe COVID-19
Petrilli et al. [33]	USA	May-22	Mar 1–Apr 8	PS	5279	2615 (49.54)	Median 54 (IQR 38–66)	30	2741/5279 (36.28)	1084/1865 (34.60)	NA	NA	NA	Disease progression
Simonnet et al. [15]	France	Apr-9	Feb 27–Apr 5	RCS	124	90 (72.58)	Median 60 (IQR 51–70)	30	85/124 (68.55)	48/59 (81.36)	29.6 (26.4–36.4)	31.1 (27.3–37.5)	27 (25.3–30.8)	IMV
Wu et al. [45]	China	Mar-27	Jan 20–Feb 19	RCS	280	151 (53.93)	Mean 43.12 (SD 19.02)	NA	83/280 (29.64)	NA	24.1 ± 3.0	25.8 ± 1.8	23.6 ± 3.2	Severe/critical COVID-19
Xiong et al. [46]	China	May-8	Jan 1–Mar 10	RCS	131	75 (57.25)	Mean 63.3 (SD 13.2)	NA	30/131 (22.90)	NA	23.1 ± 4.0	22.6 ± 3.7	23.3 ± 4.1	Severe/critical COVID-19
Zhang et al. [16]	China	May-21	Feb7-Mar 27	RCS	43	NA	Mean 24.38 (SD 4.00)	28	12/43 (27.90)	NA	23.39 (21.62–26.34)	29.32 (28.91–29.40)	23.18 (21.62–24.59)	Death
Zheng et al. [34]	China	Apr-19	Jan 17–Feb 11	RCS	66	49 (74.24)	47	25	19/66 (28.79)	17/45 (37.78)	26.5 ± 3.9	27.9 ± 3.3	25.9 ± 4.0	Severe COVID-19

All studies were published in 2020

*NA* not available, *RCS* retrospective case series, *PS* prospective study, *IQR* interquartile range, *SD* standard deviation, *IMV* invasive mechanical ventilation, *ICU* admission to the intensive care unit

### The association of BMI with severe COVID-19

**Fig. 2:**
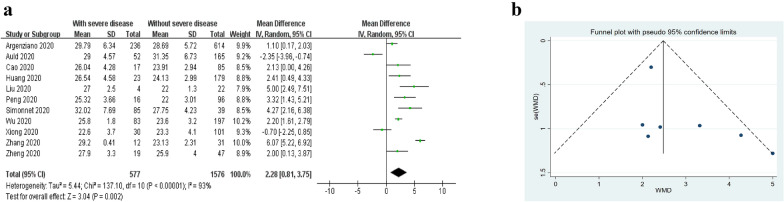
**a** Forest plot of MD in BMI between COVID-19 patients with and without severe disease. **b** Funnel plot of the included studies addressing the association between BMI and the severity of COVID-19. *MD* mean difference

The MD in BMI between COVID-19 patients with and without severe disease in the 11 studies [15–17, 34, 37, 41–46] is shown in Fig. 2. In 9 of the studies, patients with severe COVID-19 displayed a higher BMI than those with milder forms (MD ranging between 1.10 and 6.07 kg/m^2^) [15–17, 34, 37, 42–45], while in the remaining two studies, BMI was found to be higher in patients with non-severe forms of COVID-19 (MD ranging between − 0.7 and − 2.35 kg/m^2^) [41, 46]. The pooled results of these 11 studies revealed that BMI was significantly higher in patients with more severe COVID-19 (random-effects model; MD 2.28 kg/m^2^, 95% CI [0.81 to 3.75 kg/m^2^], *P* = 0.002) (Fig. 2a). The heterogeneity was high (*I*^2^ = 93%, *P* < 0.00001). Sensitivity analyses indicated that four independent studies by Auld et al. [41], Zhang et al. [16], Xiong et al. [46], and Argenziano et al. [37] were the main origins of heterogeneity. The heterogeneity weakened after the exclusion of these four studies (*I*^2^ = 30%; *P* = 0.20). No significant change was seen in the corresponding pooled OR (fixed-effects model; MD 2.48 kg/m^2^, 95% CI [2.00 to 2.96 kg/m^2^], *P* < 0.00001). The presence of publication bias was explored using funnel plot and Egger’s test, and no evidence of publication bias was found among the included studies addressing the association between BMI and the severity of COVID-19 (*P* = 0.125). No evidence of publication bias was visualized in the funnel plot (Fig. 2b).

### Pre-existing obesity and the poor outcomes of COVID-19

**Fig. 3:**
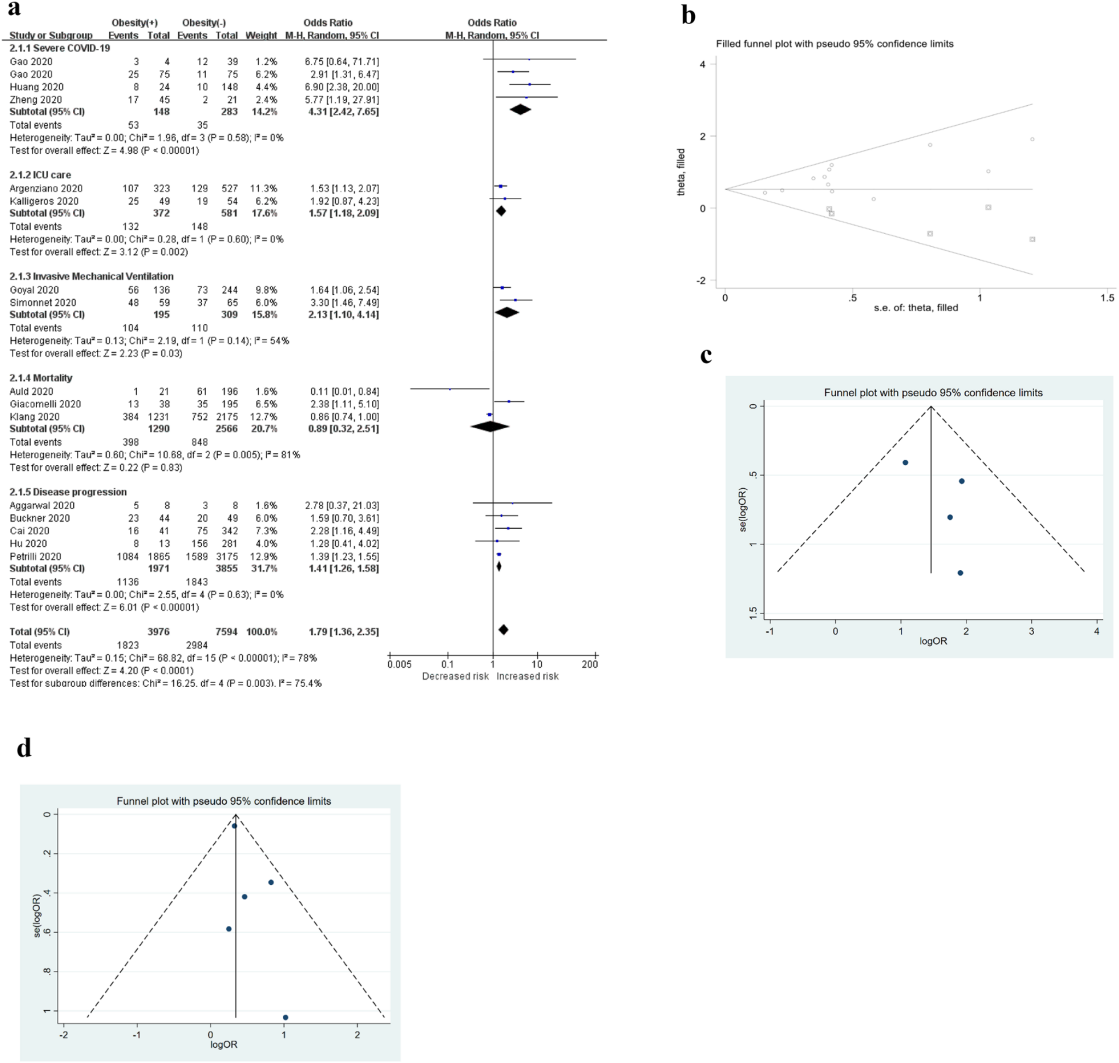
Obesity and poor composite outcomes. **a** Forest plot showed that obesity was associated with an increased risk of composite poor outcomes and its subgroup, which comprised severe COVID-19, need for ICU care, need for IMV, and disease progression in patients with COVID-19. **b** Filled funnel plot for obesity and the composite poor outcomes of COVID-19. **c** Funnel plot for obesity and severe COVID-19. **d** Funnel plot for obesity and COVID-19 progression. *ICU* intensive care unit, *IMV* invasive mechanical ventilation

A total of 16 studies [15, 32–41, 43, 47–50] were included in the analysis to determine the effect of pre-existing obesity on the poor composite outcomes of COVID-19. The pooled OR of obesity for the unfavourable outcomes of COVID-19 is summarized in Fig. 3a, which shows that the presence of obesity was associated with a 1.79 times higher risk of developing poor outcomes of COVID-19 (random-effects model; OR = 1.79, 95% CI [1.36–2.35], *P* < 0.0001); however, the heterogeneity among the different studies was quite high (*I*^2^ = 78%, *P* < 0.00001). Sensitivity analysis by excluding studies one by one showed that the studies from Petrilli et al. [33], Auld et al. [41], Klang et al. [36], and Huang et al. [43] were the major sources of heterogeneity. After the elimination of these studies, the heterogeneity disappeared (*I*^2^ = 0%, *P* = 0.57), while the association remained significant, with an OR of 1.87 (fixed-effects model; 95% CI [1.55–2.26], *P* < 0.00001). Publication bias, as assessed by Egger’s test, showed evident publication bias (*P* for Egger’s test was 0.010). Then, we used the trim-and-fill method to estimate the number of missing studies and to calculate a corrected OR as if these studies were present. The trim-and-filled method simulated 5 negative or unpublished studies that were missing from the initial analysis (OR = 1.683, 95% CI [1.408–2.011]) (Fig. 3b).

Since the reported endpoints varied among the included studies, subgroup analyses were performed to determine the impact of obesity on different endpoints of COVID-19.

#### Severe COVID-19

Subgroup meta-analysis showed that obesity was associated with severe COVID-19. The pooled OR analysis included a total of 431 patients, and 53 of 148 obese patients (35.81%) (Fig. 3a) had an increased risk of severe COVID-19, compared with 35 of 283 non-obese patients (12.37%) (fixed-effects model; OR = 4.17, 95% CI [2.32–7.48], *P* < 0.00001; *I*^2^ = 0%, *P* = 0.58). Sensitivity analysis showed that the results were not affected by any individual study. Egger’s regression asymmetry test (*P* = 0.362) and the funnel plot indicated no publication bias among these studies (Fig. 3c).

#### ICU care

Our exploratory secondary analyses suggested that obesity was associated with increased need for ICU intervention, as 132 of 372 obese patients (35.48%) had a higher need for ICU services than 148 of 581 non-obese COVID-19 patients (25.47%) (fixed-effects model; OR = 1.57, 95% CI [1.18–2.09], *P* = 0.002; *I*^2^ = 0%, *P* = 0.60) (Fig. 3a). Because only 2 studies were available for this pooled OR analysis, no sensitivity or trim-and-fill analyses were performed.

#### IMV use

The rate of mechanical lung ventilation in obese patients compared with non-obese patients was reported in two studies [15, 35] and included 504 COVID-19 patients. A total of 104 of 195 patients with obesity (53.33%) had an increased need for IMV support, compared with 110 of 309 patients without obesity (35.60%) (random-effects model; OR = 2.13, 95% CI [1.10–4.14], *P* = 0.03; *I*^2^ = 54%, *P* = 0.14) (Fig. 3a). Neither of these two studies reported the duration of mechanical ventilation. Because only 2 studies were available for this pooled OR analysis, no sensitivity or trim-and-fill analyses were performed.

#### Mortality rate

A total of three studies were included [36, 38, 41], with 3856 COVID-19 cases. Patients with obesity demonstrated a 30.85% (398/1290) mortality rate, compared to the mortality rate in patients without obesity, 33.05% (848/2566). However, the pooled OR showed that obesity was not associated with mortality (random-effects model; OR = 0.89, 95% CI [0.32–2.51], *P* = 0.83; *I*^2^ = 81%, *P* = 0.005) (Fig. 3a). The results of a sensitivity analysis indicated that none of the studies had a significant impact on the pooled estimate. The results of Egger’s test (*P* = 0.969) suggested no evidence for publication bias.

#### Disease progression

Pooling of the 5 studies [32, 33, 40, 49, 50] included in this meta-analysis resulted in a total of 5826 COVID-19 patients, 2979 of whom (51.13%) experienced disease progression and 1136 (38.13%) with obesity. A total of 1136 patients with obesity (57.64%) experienced disease progression, compared with 47.81% of non-obese patients (Fig. 3a). The pooled OR of obesity for the disease progression of COVID-19 suggested that the presence of obesity was associated with a higher risk of COVID-19 disease progression (fixed-effects model; OR: 1.41, 95% CI [1.26–1.58], *P* < 0.00001). No heterogeneity was seen among the mentioned studies (*I*^2^ = 0%, *P* = 0.63). Sensitivity analysis suggested that the results were not affected by any study. Publication bias was assessed by a funnel plot (Fig. 3d) and Egger’s test (*P* = 0.208), which showed no publication bias in the analysis.

### Meta-regression

**Fig. 4:**
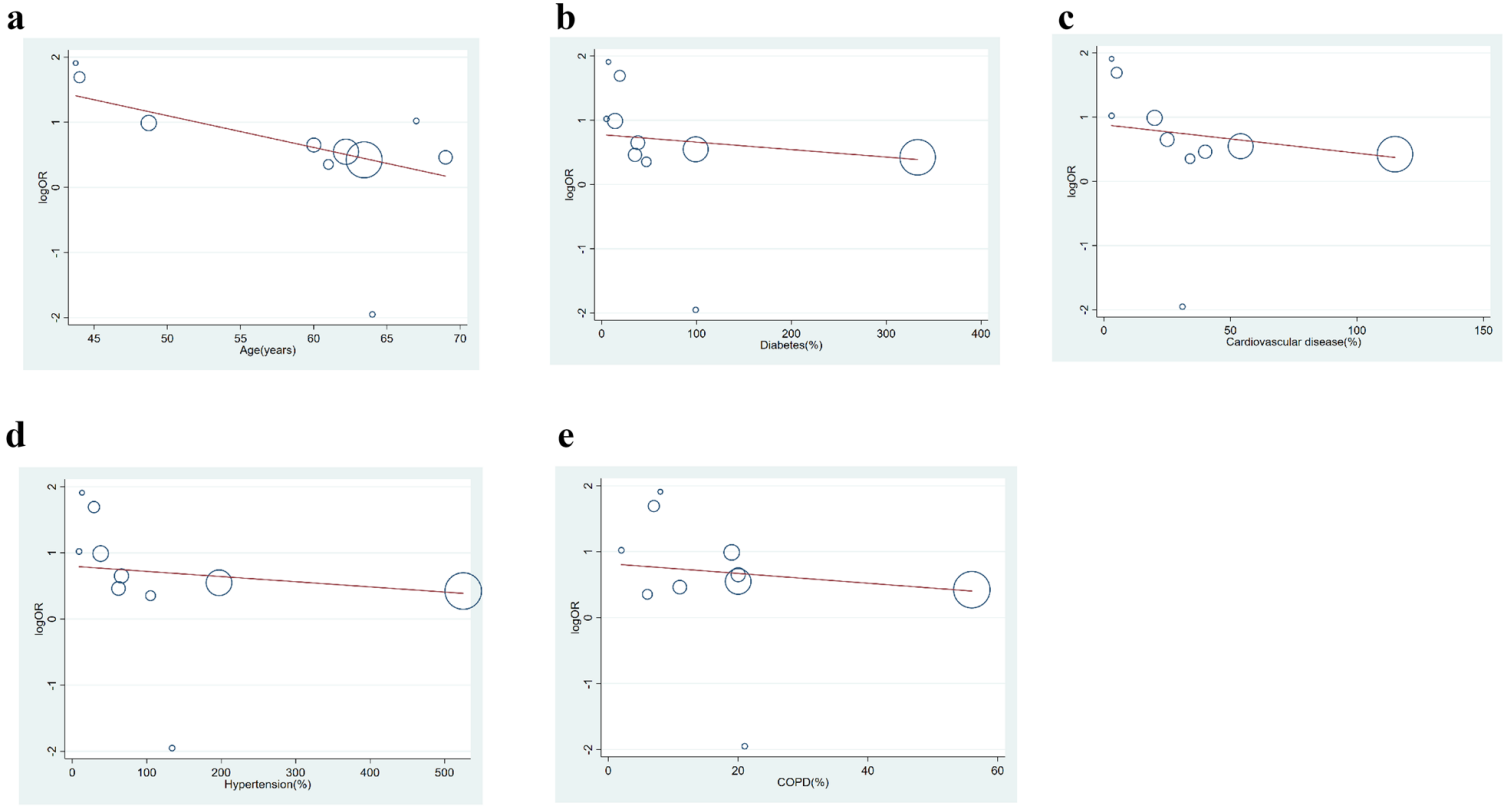
Bubble-plot for meta-regression. Meta-regression analysis showed that the association between obesity and composite poor outcome was affected by age (**a**) but not by diabetes (**b**), cardiovascular disease (**c**), hypertension (**d**), or COPD (**e**). *COPD* chronic obstructive pulmonary disease. Circles in the picture indicate studies. The red lines indicate fitted meta-regression line

Obesity is often intertwined with several chronic complications, such as hypertension, diabetes, and cardiovascular disease. To assess the influence of covariates, restricted-maximum likelihood random effects meta-regression was performed for age, diabetes, hypertension, cardiovascular disease, and COPD. Meta-regression analysis showed that the association between obesity and increased poor composite outcomes was affected by age (*P* = 0.026) (Fig. 4a) but not by diabetes (*P* = 0.249) (Fig. 4b), cardiovascular disease (*P* = 0.188) (Fig. 4c), hypertension (*P* = 0.240) (Fig. 4d), or COPD (*P* = 0.290) (Fig. 4e).

### Subgroup analyses: age

**Fig. 5:**
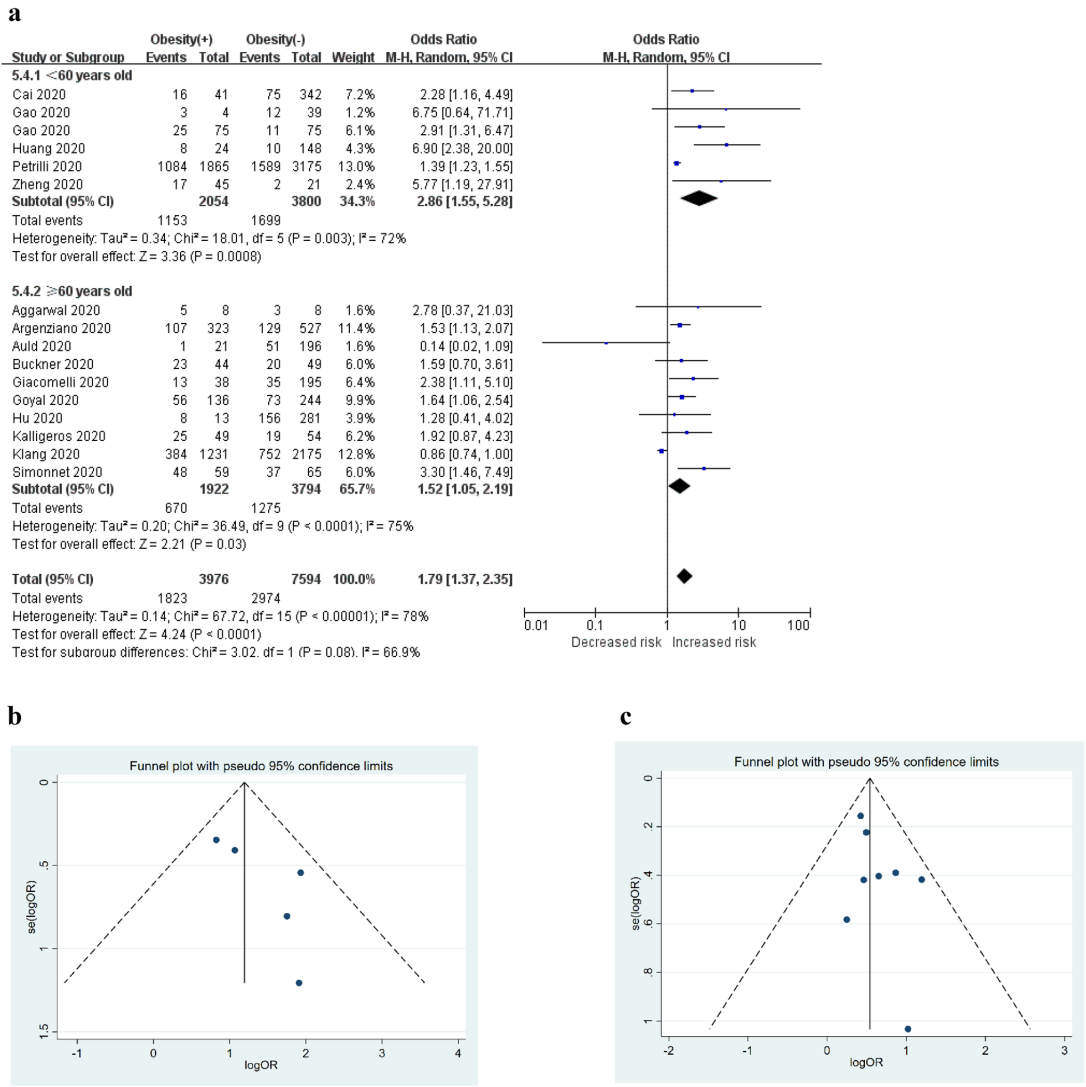
**a** Subgroup analyses based on age groups suggested that the association between obesity and poor composite outcomes was stronger in patients with a mean age < 60 years. **b** Funnel plot for obesity and composite poor outcomes of younger COVID-19 patients. **c** Funnel plot for obesity and composite poor outcomes of older COVID-19 patients

Next, we performed subgroup analysis based on age. We found that the younger group (mean age < 60 years) and the older group (mean age ≥ 60 years) differed with respect to the association between obesity and the risk of poor composite outcomes. Specifically, the younger group had a strong positive association between obesity and the poor composite outcomes of COVID-19 (random-effects model; OR = 2.86, 95% CI [1.55–5.28], *P* = 0.0008) (Fig. 5a). Heterogeneity among the included studies was severe (*I*^2^ = 72%, *P* = 0.003). Sensitivity analysis showed that the study by Petrilli et al. [33] materially changed the between-study heterogeneities. After excluding the mentioned study, the heterogeneity disappeared (*I*^2^ = 0%, *P* = 0.42), and the association was still significant (fixed-effects model; OR = 3.30, 95% CI [2.13–5.10], *P* < 0.00001). Publication bias, assessed by a funnel plot (Fig. 5b) and Egger’s test (*P* = 0.140), showed no publication bias among the studies. The association became weaker in the older group (random-effects model; OR = 1.52, 95% CI [1.05–2.19], *P* = 0.03; *I*^2^ = 75%, *P* < 0.0001) (Fig. 5a). A sensitivity analysis showed that the studies by Klang et al. [36] and Auld et al. [41] had higher heterogeneity than the remaining studies. After excluding the two studies, the heterogeneity disappeared (*I*^2^ = 0%, *P* = 0.74), and the effect of obesity on the poor composite outcomes of older COVID-19 patients was still significant, with an OR of 1.72 (fixed-effects model; 95% CI [1.40–2.11], *P* < 0.00001). The funnel plot showed a symmetrical shape for poor outcomes (Fig. 5c), and the *P* for Egger’s test was 0.178. The association between obesity and poor composite outcomes in COVID-19 was greater in younger people.

## Discussion

This systematic review and meta-analysis of 22 studies showed that obesity was associated with poor prognosis for SARS-CoV-2 infection that comprised severe COVID-19, ICU care, IMV use, and disease progression, especially among younger patients (OR 3.30 vs. 1.72). However, our meta-analysis did not find an association between obesity and hospital mortality. This result might partially be due to the extremely low proportion of deaths among COVID-19 cases with obesity in the studies we analysed. For example, a study by Auld et al. [41] reported only 1 death out of 21 cases with obesity. Meta-regression showed that the association between obesity and poor outcomes was influenced by age. Age was inversely proportional to the effect of obesity on poor outcomes. In other words, the estimated effects of obesity were lower in older patients. Subgroup analysis further demonstrated the vast difference in OR. The association between obesity and poor composite outcomes in COVID-19 was stronger in younger people. Interestingly, the effects of obesity on COVID-19 were independent of obesity-related comorbidities, such as diabetes, hypertension, and cardiovascular disease. Recently, Klang et al. [36] also found that obesity was a risk factor for the progression of COVID-19 independent of diabetes and cardiovascular disease. This suggests a significant pathophysiological link between excess adiposity and severe COVID-19 illness.

Our results on BMI and the severity of COVID-19 were similar to those of recent studies that have elucidated that BMI was significantly higher in patients with a severe form of COVID-19. Liu et al. [44] reported that BMI in severe patients was prominently higher than that in mild patients [27.0 ± 2.5 (critical group) vs. 22.0 ± 1.3 (general group), *P* < 0.001]. Peng et al. [17] conducted a retrospective analysis of 112 COVID-19 patients in Wuhan and found that the BMI of the ICU group was significantly higher than that of the general hospital admission group. After adjusting for confounding factors, each 1-unit increase in BMI was related to a 12% increase in the risk of severe COVID-19 [48]. Compared with the results of the mentioned studies, the pooled results of 11 selected studies revealed that BMI was also significantly higher in patients with severe COVID-19. Therefore, higher BMI was more common in severe or critical cases.

An increased risk of severe COVID-19 and a higher demand for ICU care were observed in patients with obesity. Obese patients had a 1.41 times higher risk of experiencing disease progression. In line with conclusions by Gao et al. [48] after adjusting for several confounders, obesity tripled the risk of COVID-19 worsening. Findings from our meta-analysis were also consistent with earlier studies on viruses such as H1N1. Several retrospective studies found that obesity increased the risk of H1N1-related hospitalization, the usage of IMV, and the death rate [51]. Possible mechanisms underlying obesity and the severity of H1N1 are relatively comprehensive. Adipose tissue acts not only as a metabolic reserve but also as an endocrine organ that induces chronic low-grade inflammation, characterized by elevated levels of proinflammatory cytokines such as leptin, interleukin (IL)-1, IL-6, IL-8, and TNF-α and decreased levels of anti-inflammatory cytokines such as adiponectin and IL-10 [52]. The constant low-grade inflammation induced by obesity results in T cell exhaustion, which impairs the immune response and the ability to eradicate virus from the host [53, 54]. Another crucial aspect of obesity is activity deficiency, which could also impair immune cell activation [55]. These results were confirmed with the use of animal models that showed that high-fat diet-induced obese (DIO) mice and leptin-deficient (OB) mice had evident lung damage, pulmonary oedema, inflammatory response, and immunopathology changes compared to wild-type (WT) mice infected with H1N1 [56]. Similarly, Zhang et al. [57] proposed heightened proinflammatory cytokines and severe pulmonary damage in DIO mice with H1N1 infection; in addition, evident leptin resistance in DIO mice impaired B cell maturation and function. Although the link between obesity and COVID-19 severity has not yet been established, several virological and physiological mechanisms that might explain the role of obesity in the pathogenesis of the disease have been suggested. Specifically, for COVID-19 patients, the SARS-CoV-2 spike protein has an increased affinity for human ACE2 compared with other mammals [58]. ACE2 is highly expressed in adipose tissue [59]. After infecting host cells, serine proteases such as TMPRSS2 mediate the cleavage of the spike and facilitate viral entry into the cells through endosomes. The virus itself causes increased apoptosis of lymphocytes, and impaired function of lymphocytes results in a fulminant cytokine storm, which is characterized by excessive circulating levels of IL-6, IL-2, IL-7, TNFα and so on [60]. IL-6, for example, is elevated in obese COVID-19 patients and has been suggested to be a key proinflammatory factor that triggers the inflammatory storm [61]. In line with this, the IL-6 blocker tocilizumab has been proposed as a treatment in COVID-19 and could increase the lymphocyte blood count of COVID-19 patients [62]. Adaptive immunity is also disrupted in obesity, with a sharply decrease in anti-inflammatory CD4+ and CD8+ cells and an increased percentage of proinflammatory immune cells such as Th17 and Th22 cells [62]. Additionally, because of the large volume of adipose tissue, the population with obesity had a significantly large amount of ACE2 and was inclined to host and stock a huge amount of virus, which resulted in increased viral shedding, immune inactivation, and cytokine storm [63]. In brief, the mentioned unfavourable chronic inflammation, dysfunction of the immune system, and higher ACE2 concentration in adipose tissue might partly explain the high risk of poor outcomes in obese COVID-19 patients. However, a more accurate understanding of the underlying mechanisms is needed.

Interestingly, the effect estimated for subgroup analysis of age ≥ 60 and < 60 years differed significantly, and obesity had a stronger impact on the younger group than on the older group (OR 3.30 vs. 1.72). The results were entirely consistent with the latest three studies, which both indicated that younger patients with obesity were crucial high-risk populations [16, 36, 64]. Lighter et al. [64] divided COVID-19 patients into two groups, a younger group (less than 60 years) and an older group (more than 60 years), and suggested that obesity was associated with disease progression only in the younger group. Zhang et al. and Klang et al. [16, 36] found that obesity was a risk factor for mortality in young patients. In a cross-sectional study conducted by Bhasin et al. [65], patients less than 50 years infected with SARS-CoV-2 had a higher mean BMI, and BMI appeared to decrease with increasing age among COVID-19 patients, which is consistent with Kass’s study [9]. The results suggested that obesity might be particularly important among younger COVID-19 patients. Reasons for this association are undefined. The findings of Deng et al. [66] suggested that visceral adiposity, such as liver fat deposition, epicardial adipose tissue, and perirenal fat accumulation, can predict the risk of young obese patients with severe COVID-19. Visceral fat accumulation can contribute to insulin resistance and hyperglycaemia, all measurable predictors of COVID-19 complications [67]. In addition, at a younger age, obesity-related sleep apnoea might be a risk factor for the progression of COVID-19 through atelectatic burden and hypoxemia [65]. Specifically, elderly patients were more likely to have hypertension and cardiovascular disease. However, patients with these comorbidities are likely to be treated with angiotensin-converting enzyme inhibitors (ACEIs) and angiotensin receptor blockers (ARBs). A meta-analysis of 12 studies indicated that a lower risk of mortality was observed among COVID-19 patients who were taking ACEIs/ARBs for the treatment of hypertension [68]. To some extent, ACEIs/ARBs treatment may alleviate the progression of COVID-19 in the older group. Further epidemiological and mechanistic studies to clarify the poor outcomes of younger obese patients are needed. Much attention has been given to elderly patients with multiple comorbidities, but from the above evidence, younger patients with obesity should be considered a higher risk group for COVID-19. Reducing the threshold for SARS-CoV-2 testing and greater alertness should be maintained in this at-risk population.

Importantly, the need for IMV increased in obese patients. Pulmonary function studies have concluded that structural changes in the thoracic-abdominal region in patients with obesity limit the mobility of the diaphragm, which is essential for adequate pulmonary function [69]. Obesity-related impaired lung function could partly explain why obese COVID-19 patients more often need IMV. COVID-19 patients with severe obesity present evident management challenges with regard to ventilation support [70].

Nevertheless, we noticed no significant difference in mortality, which seemed to be paradoxical and unaccountable. The effect of obesity on the mortality of ARDS was controversial in previous studies [20, 71, 72]. An obesity survival paradox has been observed in patients with pneumonia. That is, despite the increased risk of pneumonia and difficulties of IMV, the risk of death in patients with obesity and pneumonia might be decreased [73]. Several pathophysiological mechanisms might partially explain the possible association between obesity and lower hospital mortality of severe illness, including higher serum levels of cholesterol binding endotoxin and excess energy stored in adipose tissues [73, 74] or other unidentified factors. However, the evident heterogeneity among the three included studies that involved information about the mortality of COVID-19 patients in our systematic review and meta-analysis might lead to the unaccountable results. Moreover, one of the three studies included only reported ICU mortality, which may lead to an underestimation of mortality, because the patients may have died in the general ward after leaving the ICU or might have been readmitted to the ICU and died during this process. Whether the obesity paradox has been broken by COVID-19 could not be confirmed. These conflicting findings still need larger studies, in particular, prospective studies designed to analyze BMI and other clinical information in all patients, to further determine the precise roles of obesity in mortality of patients with COVID-19.

### Limitations

Several limitations also exist in our study: (a) one major drawback that merits consideration is the inherent high heterogeneity across studies. The definitions of obesity varied (BMI from 28 to 30 kg/m^2^). Additionally, imprecise measurements of BMI (which often were estimations or from patient-reported data). In addition, the study designs were different. Additionally, there was large variation in the sample size among studies (16 to 5279). (b) Our study did not have sufficient data for subgroup analysis of normal weight and overweight, since most included studies mainly focused on obese and non-obese individuals, which might ignore the effect of overweight on poor outcomes of COVID-19 patients. (c) Several studies were preprints.

## Conclusions

In summary, our systematic review and meta-analysis assessed the effect of obesity on the severity of COVID-19 and suggested that obesity was strongly associated with poor composite outcomes of COVID-19, including an increased need for ICU care and IMV support, a higher risk of severe COVID-19, and disease progression. Younger patients with high BMI seemed to be at particularly high risk, and this population should be treated with increased precaution, priority in detection and testing, and aggressive therapy. These results are of great importance and may be helpful for public health strategy-making, especially in some countries affected by a high morbidity of obesity. Obesity might not be associated with hospital mortality in COVID-19 patients. Efforts to understand the impact of obesity on the mortality of COVID-19 patients should be a research priority in the future.

## Supplementary information

**Additional file 1: Table S1.** MOOSE checklist. **Table S2.** Search strategy for each database. **Table S3.** Newcastle–Ottawa quality assessment scale for identified studies.

## Data Availability

All data and materials can be accessed via CYN and SJR.
