# Supplementary material for: Obesity is associated with increased severity of disease in COVID-19 pneumonia: a systematic review and meta-analysis
Source: Eur J Med Res. 2020 Dec 2;25:64. doi: 10.1186/s40001-020-00464-9 (PMC7708895; doi:10.1186/s40001-020-00464-9)
Supplement: Supplementary file 1 — Additional file 1: Table S1. MOOSE checklist. Table S2. Search strategy for each database. Table S3. Newcastle–Ottawa quality assessment scale for identified studies. [file 40001_2020_464_MOESM1_ESM.docx]

**Obesity is associated with increased severity of disease in COVID-19 pneumonia: A systematic review and meta-analysis**

Table S1 MOOSE Checklist

| **Criteria** | | **Brief description of how the criteria were handled in the meta-analysis** |
| --- | --- | --- |
| **Reporting of background** | |  |
| 1 | Problem definition | Since the conflicting evidence, limitations of past reviews, and availability of new data, this study was aimed at investigating the association between obesity and poor outcomes of COVID-19 by performing a systematic review and meta-analysis. |
| 2 | Hypothesis statement | Obesity was associated with poor outcomes of COVID-19. |
| 3 | Description of study outcomes | Poor outcomes including severe COVID-19, need for ICU care, IMV, mortality, and disease progression. |
| 4 | Type of exposure | obesity |
| 5 | Type of study designs used | All studies were observational studies. |
| 6 | Study population | (a) adult COVID-19 patients;(b) the information about BMI or the prevalence of obesity; (c) measured outcomes using severe COVID-19, need for ICU care, IMV, mortality, or disease progression. |
| **Reporting of search strategy should include** | |  |
| 7 | Qualifications of searchers | YJX and SJR |
| 8 | Search strategy, including time period included in the synthesis and keywords | Time period: articles published between November 1, 2019 and May 24, 2020 were eligible for inclusion.  Search strategy: Table S2 |
| 9 | Databases and registries searched | PubMed, EMBASE, and Web of science |
| 10 | Search software used, name and version, including special features | Google Chrome was used to search PubMed, EMBASE and Web of science.  Endnote X9 was used to manage references. |
| 11 | Use of hand searching | The reference lists of all potential studies were retrieved aimed at identifying additional suitable articles. |
| 12 | List of citations located and those excluded, including justifications | The literature search identified 1163 studies. Of these, studies were excluded for the following reasons: unique records (611), records were excluded after screening their titles and abstracts (427), unobtainable articles (6), had no information about BMI or obesity (5), did not divided groups by outcomes (12), had subjects similar with another record (1). |
| 13 | Method of addressing articles published in languages other than English | We placed no restrictions on language; two articles in Chinese could be located and were understandable. |
| 14 | Method of handling abstracts and unpublished studies | No searching of the grey literature performed |
| 15 | Description of any contact with authors | None |
| **Reporting of methods should include** | |  |
| 16 | Description of relevance or appropriateness of studies assembled for assessing the hypothesis to be tested | Table of included studies (Table 1) |
| 17 | Rationale for the selection and coding of data | We extracted data of BMI and the prevalence of obesity to compare poor outcomes between COVID-19 patients with obesity and without obesity. |
| 18 | Assessment of confounding | NA |
| 19 | Assessment of study quality, including blinding of quality assessors; stratification or regression on possible predictors of study results | Table S3 |
| 20 | Assessment of heterogeneity | Heterogeneity among studies was evaluated with the I^2^ statistic: 25%, 50%, and 75% represented low, moderate, and high degrees of heterogeneity, respectively. |
| 21 | Description of statistical methods in sufficient detail to be replicated | Data were assembled and analyzed by Review Manager (RevMan) Version 5.3 (The Nordic Cochrane Center, Copenhagen, Denmark) and Stata version 15 software. The chosen of proper effect model was based on the analysis results: if significant heterogeneity was not present (I^2^≤50%), we used fixed-effects model to pool outcomes; random-effects model was utilized if I^2^＞50%. |
| 22 | Provision of appropriate tables and graphics | See Figure 1 and Table 1 |
| **Reporting of results should include** | |  |
| 23 | Graph summarizing individual study estimates and overall estimate | Figure 1 |
| 24 | Table giving descriptive information for each study included | Table 1 |
| 25 | Results of sensitivity testing | Sensitivity testing was conducted by Stata. And the detailed description of sensitivity results was in the results of manuscript. |
| 26 | Indication of statistical uncertainty of findings | If there was statistical heterogeneity, further sensitivity analyses were performed by excluding studies one by one. After the significant clinical heterogeneities vanished, the fixed-effect model was used for meta-analysis. |
| **Reporting of discussion should include** | |  |
| 27 | Quantitative assessment of bias | If a meta-analysis included more than three studies, the possible publication bias was assessed by funnel plot, Egger’s test, and the trim and fill method. And the funnel plots were presented in Figure S1. |
| 28 | Justification for exclusion | All studies were excluded based on the pre-defined inclusion criteria. |
| 29 | Assessment of quality of included studies | Most included articles in our meta-analysis were in good quality. In order to amplify the sample and increase the credibility and extensibility of the results, we included all of the articles. |
| **Reporting of conclusions should include** | |  |
| 30 | Consideration of alternative explanations for observed results | The discussion of other reasons for the association between obesity and mortality was made in the discussion. |
| 31 | Generalization of the conclusions | Generalized in the conclusions. |
| 32 | Guidelines for future research | Obesity may be not associated with COVID-19 patients’ mortality. Efforts to understand the impact of obesity on mortality of COVID-19 patients should be a research priority. |
| 33 | Disclosure of funding source | **Funding/Support:** This research did not receive any specific grant from funding agencies in the public, commercial, or not-for-profit sectors.  The authors declared no conflict of interest. |

From: Stroup DF, Berlin JA, Morton SC, et al. Meta-analysis of observational studies in epidemiology: a proposal for reporting. Meta-analysis Of Observational Studies in Epidemiology (MOOSE) group. *JAMA.* 2000;283(15):2008-2012.

Table S2 Search Strategy for Each Database.

| **Literature databases** | **Search items** | **Items found** |
| --- | --- | --- |
| PUBMED | #1  "COVID-19"[Supplementary Concept] OR ((((((((((("2019 novel coronavirus disease"[Title/Abstract] OR "2019 novel coronavirus disease"[Title/Abstract]) OR "covid 19 pandemic"[Title/Abstract]) OR "sars cov 2 infection"[Title/Abstract]) OR (((((((("COVID-19"[All Fields] OR "covid 2019"[All Fields]) OR "severe acute respiratory syndrome coronavirus 2"[Supplementary Concept]) OR "severe acute respiratory syndrome coronavirus 2"[All Fields]) OR "2019-nCoV"[All Fields]) OR "SARS-CoV-2"[All Fields]) OR "2019ncov"[All Fields]) OR (("wuhan"[All Fields] AND ("coronavirus"[MeSH Terms] OR "coronavirus"[All Fields])) AND (2019/12/1:2019/12/31[Date - Publication] OR 2020/1/1:2020/12/31[Date - Publication]))) AND "virus disease"[Title/Abstract])) OR "2019 novel coronavirus infection"[Title/Abstract]) OR "2019 ncov infection"[Title/Abstract]) OR "coronavirus disease 2019"[Title/Abstract]) OR "coronavirus disease 19"[Title/Abstract]) OR "2019 ncov disease"[Title/Abstract]) OR "covid 19 virus infection"[Title/Abstract]) OR "covid 19 virus infection"[Title/Abstract]))  #2  "obesity"[Title/Abstract]  #1 AND #2  OR  #1  "COVID-19"[Supplementary Concept] OR ((((((((((("2019 novel coronavirus disease"[Title/Abstract] OR "2019 novel coronavirus disease"[Title/Abstract]) OR "covid 19 pandemic"[Title/Abstract]) OR "sars cov 2 infection"[Title/Abstract]) OR (((((((("COVID-19"[All Fields] OR "covid 2019"[All Fields]) OR "severe acute respiratory syndrome coronavirus 2"[Supplementary Concept]) OR "severe acute respiratory syndrome coronavirus 2"[All Fields]) OR "2019-nCoV"[All Fields]) OR "SARS-CoV-2"[All Fields]) OR "2019ncov"[All Fields]) OR (("wuhan"[All Fields] AND ("coronavirus"[MeSH Terms] OR "coronavirus"[All Fields])) AND (2019/12/1:2019/12/31[Date - Publication] OR 2020/1/1:2020/12/31[Date - Publication]))) AND "virus disease"[Title/Abstract])) OR "2019 novel coronavirus infection"[Title/Abstract]) OR "2019 ncov infection"[Title/Abstract]) OR "coronavirus disease 2019"[Title/Abstract]) OR "coronavirus disease 19"[Title/Abstract]) OR "2019 ncov disease"[Title/Abstract]) OR "covid 19 virus infection"[Title/Abstract]) OR "covid 19 virus infection"[Title/Abstract]))  #2  "characteristics"[Title/Abstract]  #1 AND #2 | 200 |
| Web of Science | **#1**  TS=("COVID-19"[Supplementary Concept] OR 2019 novel coronavirus disease OR COVID19 OR COVID-19 pandemic OR SARS-CoV-2 infection OR COVID-19 virus disease OR 2019 novel coronavirus infection OR 2019-nCoV infection OR coronavirus disease 2019 OR coronavirus disease-19 OR 2019-nCoV disease OR COVID-19 virus infection OR severe acute respiratory syndrome coronavirus 2) 数据库= WOS, BCI, CSCD, DIIDW, FSTA, KJD, MEDLINE, RSCI, SCIELO, ZOOREC 时间跨度=2019-2020  **#2**  TS=("obesity"[MeSH Terms] OR overweight OR adiposity)  数据库= WOS, BCI, CSCD, DIIDW, FSTA, KJD, MEDLINE, RSCI, SCIELO, ZOOREC 时间跨度=2019-2020  **#1 AND #2**  **OR**  **#1**  TS=("COVID-19"[Supplementary Concept] OR 2019 novel coronavirus disease OR COVID19 OR COVID-19 pandemic OR SARS-CoV-2 infection OR COVID-19 virus disease OR 2019 novel coronavirus infection OR 2019-nCoV infection OR coronavirus disease 2019 OR coronavirus disease-19 OR 2019-nCoV disease OR COVID-19 virus infection OR severe acute respiratory syndrome coronavirus 2) 数据库= WOS, BCI, CSCD, DIIDW, FSTA, KJD, MEDLINE, RSCI, SCIELO, ZOOREC 时间跨度=2019-2020  **#2**  TS=(clinical characteristics)  数据库= WOS, BCI, CSCD, DIIDW, FSTA, KJD, MEDLINE, RSCI, SCIELO, ZOOREC 时间跨度=2019-2020  **#1 AND #2** | 638 |
| EMBASE | **#1**  'covid 19':ab,ti OR '2019 novel coronavirus disease':ab,ti OR covid19:ab,ti OR 'covid-19 pandemic':ab,ti OR 'sars-cov-2 infection':ab,ti OR 'covid-19 virus disease':ab,ti OR '2019 novel coronavirus infection':ab,ti OR '2019-ncov infection':ab,ti OR 'coronavirus disease 2019':ab,ti OR 'coronavirus disease-19':ab,ti OR '2019-ncov disease':ab,ti OR 'covid-19 virus infection':ab,ti OR 'severe acute respiratory syndrome coronavirus 2':ab,ti  **#2**  'obesity'/exp  **#1 AND #2**  **OR**  **#1**  'covid 19':ab,ti OR '2019 novel coronavirus disease':ab,ti OR covid19:ab,ti OR 'covid-19 pandemic':ab,ti OR 'sars-cov-2 infection':ab,ti OR 'covid-19 virus disease':ab,ti OR '2019 novel coronavirus infection':ab,ti OR '2019-ncov infection':ab,ti OR 'coronavirus disease 2019':ab,ti OR 'coronavirus disease-19':ab,ti OR '2019-ncov disease':ab,ti OR 'covid-19 virus infection':ab,ti OR 'severe acute respiratory syndrome coronavirus 2':ab,ti  **#2**  'clinical characteristics':ab,ti  **#1 AND #2** | 311 |
| Overall |  | 1149 |

Table S3 Newcastle-Ottawa quality assessment scale for identified studies.

| First author, year | Selection | Comparability | Exposure/Outcome | Total Score |
| --- | --- | --- | --- | --- |
| Aggarwal 2020 | **🟑🟑****🟑** | **🟑** | **🟑🟑** | 6 |
| Argenziano 2020 | **🟑🟑🟑** | **🟑** | **🟑🟑** | 6 |
| Auld 2020 | **🟑🟑🟑** | **🟑** | **🟑🟑** | 6 |
| Buckner 2020 | **🟑🟑** | **🟑** | **🟑🟑🟑** | 6 |
| Cao 2020 | **🟑****🟑** | **🟑** | **🟑****🟑** | 5 |
| Gao 2020 | **🟑🟑🟑** | **🟑** | **🟑🟑** | 6 |
| Feng Gao 2020 | **🟑🟑🟑** | **🟑** | **🟑🟑** | 6 |
| Giacomelli 2020 | **🟑🟑** | **🟑** | **🟑🟑🟑** | 6 |
| Goyal 2020 | **🟑🟑🟑** | **🟑** | **🟑🟑** | 5 |
| Hu 2020 | **🟑🟑** | **🟑** | **🟑🟑** | 5 |
| Huang 2020 | **🟑🟑🟑** | **🟑** | **🟑🟑** | 6 |
| Klang 2020 | **🟑🟑🟑** | **🟑** | **🟑🟑** | 6 |
| Kalligeros 2020 | **🟑🟑🟑** | **🟑** | **🟑🟑** | 6 |
| Peng 2020 | **🟑🟑🟑** | **🟑** | **🟑🟑** | 6 |
| Petrilli 2020 | **🟑🟑** | **🟑** | **🟑🟑** | 5 |
| Simonnet 2020 | **🟑🟑🟑** | **🟑** | **🟑🟑** | 6 |
| Wu 2020 | **🟑🟑🟑** | - | **🟑🟑** | 5 |
| Xiong 2020 | **🟑****🟑** | - | **🟑🟑** | 4 |
| Zhang 2020 | **🟑🟑🟑** | **🟑** | **🟑🟑** | 6 |
| Zheng 2020 | **🟑🟑🟑** | - | **🟑🟑** | 5 |
| Cai 2020 | **🟑🟑** | **🟑** | **🟑🟑🟑** | 6 |
| Liu 2020 | **🟑🟑** | **🟑** | **🟑🟑** | 5 |

Assessment of overall quality with Newcastle Ottawa Scale (NOS). A study was considered of good quality if there are 3 or 4 stars in selection domain, and 1 or 2 stars in comparability domain, and 2 or 3 stars in exposure /outcome domain.
